# Differential Interpretation of Mountain Temperatures by Endospermic Seeds of Three Endemic Species Impacts the Timing of In Situ Germination

**DOI:** 10.3390/plants9101382

**Published:** 2020-10-16

**Authors:** Marco Porceddu, Hugh W. Pritchard, Efisio Mattana, Gianluigi Bacchetta

**Affiliations:** 1 Sardinian Germplasm Bank (BG-SAR), Hortus Botanicus Karalitanus (HBK), University of Cagliari, Viale S. Ignazio da Laconi, 9-11, 09123 Cagliari, Italy; bacchet@unica.it; 2 Centre for the Conservation of Biodiversity (CCB), Life and Environmental Sciences Department, University of Cagliari, Viale S. Ignazio da Laconi 11-13, 09123 Cagliari, Italy; 3 Royal Botanic Gardens, Kew, Wellcome Trust Millennium Building, Wakehurst, Ardingly, West Sussex RH17 6TN, UK; h.pritchard@kew.org (H.W.P.); E.Mattana@kew.org (E.M.)

**Keywords:** embryo growth, global warming, Grossulariaceae, IPCC scenarios, morphophysiological dormancy, Paeoniaceae, phenology, Ranunculaceae, soil temperature, tree canopy

## Abstract

Predicting seed germination in the field is a critical part of anticipating the impact of climate change on the timing of wild species regeneration. We combined thermal time and soil heat sum models of seed germination for three endemic Mediterranean mountain species with endospermic seeds and morphophysiological dormancy: *Aquilegia barbaricina*, *Paeonia corsica*, and *Ribes sandalioticum*. Seeds were buried in the soil within the respective collection sites, both underneath and outside the tree canopy, and their growth was assessed regularly and related to soil temperatures and estimates of the thermal characteristics of the seeds. The thermal thresholds for embryo growth and seed germination of *A. barbaricina* assessed in previous studies under controlled conditions were used to calculate soil heat sum accumulation of this species in the field. Thermal thresholds of seed germination for *P. corsica* and *R. sandalioticum* were not previously known and were estimated for the first time in this field study, based on findings of previous works carried out under controlled conditions. Critical embryo length and maximum germination for *A. barbaricina* were reached in April, and in December for *R. sandalioticum*. Seeds of *P. corsica* stay dormant in the ground until the following summer, and the critical embryo length and highest germination were detected from September to December. Soil heat sum models predicted earlier germination by one month for all three species under two Intergovernmental Panel on Climate Change (IPCC) scenarios, based on the assumption that the estimated thermal thresholds will remain constant through climate changes. This phenological shift may increase the risk of mortality for young seedlings. The models developed provide important means of connecting the micro-environmental niche for in situ seed germination and the macro-environmental parameters under a global warming scenario.

## 1. Introduction

The Mediterranean climate is characterized by a high seasonality in temperature and precipitation, which leads to a hot drought in summer and a cool, wet winter [1]. These conditions impact the timing of plant emergence, since dry summer conditions limit water availability and thus seed germination and growth, while cool winter temperatures can limit germination, even when the season has high water availability [2,3,4]. In seasonal climates and in moist soils, temperature is usually the main environmental factor influencing seed germination [5], which is a complex adaptive trait that determines plant establishment and contributes to population persistence [6]. Moreover, this adaptive trait becomes even more complex in species that produce dormant seeds, by which germination is prevented before or during unfavourable environmental conditions for emergence and subsequent seedling development [7]. Dormancy breaking and germination requirements are specific for each species and depend on phylogeny, geographical distribution, habitat preference, life cycle and type of dormancy [8]. Two common dormancy types are morphological (MD) and physiological (PD), and these may also be found in combination (e.g., morphophysiological dormancy, MPD) [5,7]. MPD is frequent in parts of the world with moist seasonal climates [5], and breaking MPD requires the initial growth of the embryo within the seed and an environmental treatment, often exposure to a precise temperature, to overcome the physiological block to radicle emergence [7,9]. Thus, morphological and/or physiological changes may occur as dormant seeds gain the ability to germinate at some particular time(s) during the year [7]. Generally, in seeds with MPD, the seed can germinate after the embryo has grown to a critical size and developed morphologically inside the seed, and the physiological limitations of the embryo been overcome. In some species, embryo growth and dormancy break are promoted by the same environmental conditions, while in others they require different conditions [7]. Therefore, to understand the performance of the seeds in the field, information is needed on the various changes that occur in the seeds and how they are correlated with the annual climatic cycle in situ [10].

In non-dormant seeds, the germination response to accumulated temperature has been modelled using a thermal time (*θ*) approach [11,12,13,14,15,16,17]. In this model, seeds accumulate units of thermal time to germinate for a percentile *g* of the population, and when some dormancy is present seed germination may be predicted in relation to thermal time accumulation above a gradually changing base temperature (*T*_b_) [18]. Furthermore, as reported recently by Porceddu et al. [19] for *Aquilegia barbaricina* Arrigoni & E.Nardi, this approach may be applied also to identify the thermal thresholds (*T*_b_ and *θ*) requirements for embryo growth within the seed. Thermal time models have been shown to be robust and have many purposes, including predicting seed germination in the field (i.e., [20,21,22]), assessing the impact of different simulated climate warming scenarios on seed dormancy release and germination [23,24], and identifying the role of diurnally alternating temperatures in seed responses to climate change [25]. Porceddu et al. [26] used a soil heat sum model to predict *in situ* seed germination of *Rhamnus persicifolia* Moris, both underneath and outside an *Alnus glutinosa* (L.) Gaertn. canopy. Similarly, Ordoñez-Salanueva et al. [27] used this approach to examine if the shrub canopy might maintain a favourable temperature for seed germination of *Polaskia chende* (Rol-.Goss.) A.C. Gibson & K.E. Horak and *Polaskia chichipe* (Rol.-Goss.) Backeb under predicted climate change scenarios.

The Intergovernmental Panel on Climate Change (IPCC) has predicted temperature increases of approx. 2–4 °C by 2090–2099 [28]. In the Fifth Assessment Synthesis Report [29], the IPCC integrated and described four different Representative Concentration Pathways (RCPs), including two intermediate scenarios (RCP4.5 and RCP6.0) and one scenario with very high (RCP8.5) anthropogenic greenhouse gas emissions. The increase of global mean surface temperature by the end of the 21^st^ century (2081–2100) is likely to be 1.1–3.1 °C under RCP4.5 and RCP6.0 and 2.6–4.8 °C under RCP8.5 [29].

Large increases in temperature have been predicted and reported for the Mediterranean mountain ranges [30,31]. A characteristic trend of Mediterranean mountains is that warming is generally coupled with a reduction in precipitation mainly in summer [31]. This trend makes Mediterranean mountains particularly sensitive to climate change [32]. The effects of the temperature rise on the reproductive success of plant species may be anticipated in Mediterranean mountains [32], and these changes can be considered in the context of global warming scenarios [33]. In addition, Mediterranean mountains represent one of the most important centres of biodiversity and differentiation of the world [34]. The mountains of Central Northern Sardinia (Italy), precisely Supramontes and Gennargentu massif, have been recognised as two of the biodiversity micro-hotspots within Sardinian region [35]. In detail, Supramontes and Gennargentu largely coincide with two of the richest floristic territories for endemics and are considered two of the main massifs of Sardinia, with mountains reaching the highest altitudes in the Island [36,37]. Although in mountainous areas climate warming is expected to shift species’ ranges towards higher altitudes [38], for some species growing in these areas the altitudinal shifts of their distribution may be more complex. For example, in mountain species located at the edge of its distributional and ecological range, such as *Gentiana lutea* subsp. *lutea* L. in Sardinia, the effect of global warming would tend to reduce the altitudinal range towards higher elevations, increasing the risk of local extinctions due their isolation and restriction to marginal habitats [39]. Therefore, to better understand the impact of global warming on seed germination and seedling emergence of species growing in restricted areas of Mediterranean mountains (which may coincide with its elevational limits) is fundamental.

Riparian vegetation present in Supramontes and Gennargentu massif is dominated principally by *Alnus glutinosa* (L.) Gaertn. with associated taxa such as *Taxus baccata* L., *Ilex aquifolium* L. and *R. persicifolia*. The canopies of woody plants modify the microclimate beneath and around them through interception of precipitation and by shading, which influences maximum soil temperatures [40]. Lower air and soil temperatures below canopies could be critical for seedlings to withstand the summer drought, consequently suggesting that shade constitutes a key facilitative mechanism in Mediterranean systems, even without improving soil water conditions [41].

In this work, we focused our attention on three rare and threatened Sardinian endemic species growing in the upper part of the mountains of Gennargentu massif and Supramontes, such as *Aquilegia barbaricina*, *Paeonia corsica* Sieber *ex* Tausch, and *Ribes multiflorum* Kit *ex* Roem & Schult. subsp. *sandalioticum* Arrigoni. These taxa frequently grow in the same ecosystem and under ecological conditions and close to the canopy of woody plants. Seeds of *A. barbaricina*, *P. corsica*, and *R. multiflorum* subsp. *sandalioticum* (hereafter *R. sandalioticum*) are endospermic and contain a linear underdeveloped embryo (*sensu* [42]). The seeds of these species show morphophysiological dormancy (MPD) [19,43,44]. Warm (25 °C for three months) followed by a cold stratification (5 °C for 3 months) release dormancy in *A. barbaricina* [19], while warm stratification (25 °C for three months) is required for dormancy break in seeds of *P. corsica* and *R. sandalioticum* [43,44]. Although these species were thoroughly studied under laboratory controlled conditions [19,43,44], how the seed germination kinetics of these species interact with their natural habitat (e.g., favourable environmental conditions for seed dormancy release, embryo growth, and radicle protrusion) needs to be investigated in order to supplement the limited knowledge present in the literature about the thermal control of MPD-type seeds. Thermal threshold requirements for embryo growth and seed germination for *A. barbaricina* have been previously estimated in controlled conditions [19] and the base temperature and thermal time values were used here to predict and validate the seeds *in situ* germination. Thermal thresholds for seed germination of *P. corsica* and *R. sandalioticum* were not previously known and were estimated based on findings of studies previously carried out under controlled conditions [43,44] and field observations in this study. The study comprises two representative localities for the species under study: Rio Correboi (located in the Gennargentu massif) for *A. barbaricina* and *P. corsica*, and Monte Novo San Giovanni (located in Supramontes) for *R. sandalioticum*.

In this work, embryo morphology, seed germination, and thermal requirements of *A. barbaricina, P. corsica*, and *R. sandalioticum* were correlated with the environmental temperature conditions, in order to: (1) investigate the field embryo growth, seed germination and epicotyl emergence of these Sardinian endemic mountain species with endospermic seeds and morphophysiological dormancy; (2) develop thermal time and soil heat sum models to predict their seed germination phenology in the field, under present climatic conditions and two different IPCC scenarios of increasing temperatures, under the assumption that the estimated thermal thresholds will remain constant through climate changes. We expected that the studied species growing in the same habitat and ecosystem may respond to current and predicted mountain temperature differently by synchronizing the time and the rate of embryo growth and radicle protrusion in a period of time favourable for the subsequent seedling development in situ.

## 2. Results

### 2.1. Embryo Growth and Germination Tests in Natural Conditions

**Figure 1:**
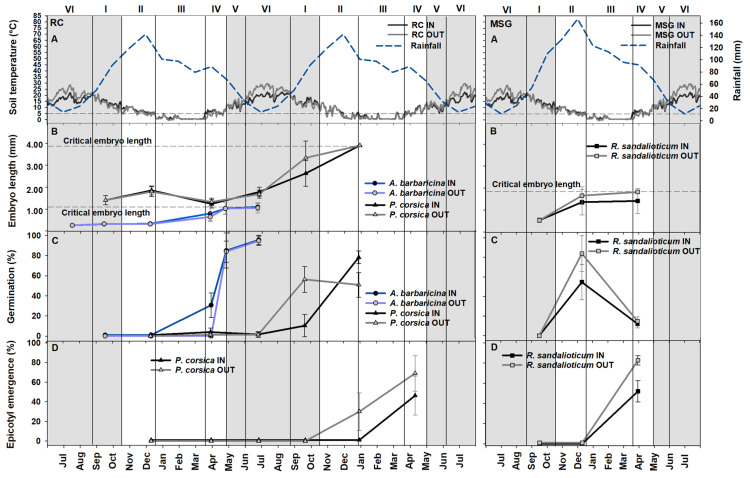
(**A**) Annual trends of mean daily temperatures recorded in the soil both underneath (IN) and outside (OUT) the tree canopy for Rio Correboi (RC) and Monte Novo San Giovanni (MSG), and mean monthly rainfall obtained from the nearby weather stations of Fonni for RC and of Montes for MSG from June 2011 to August 2013 and from June 2011 to August 2012 for RC and MSG, respectively. (**B**) Embryo length in mm (20 seeds at each exhumation time). (**C**) Field germination (3 replicates of 25 seeds each) IN and OUT at each time of exhumation. (**D**) Field epicotyl emergence (3 replicates of 25 seeds each) IN and OUT at each time of exhumation of *P. corsica* and *R. sandalioticum*. The background grey squares correspond to the presence of the tree canopy. I, II, III, IV, V and VI correspond to different periods.

Soil temperatures recorded by data loggers were very similar for the two localities (RC, “Rio Correboi”; MSG, “Monte Novo San Giovanni”; Figure 1A), with an annual mean temperature of ca. 9.5 °C for IN (i.e., underneath the tree canopy) in both populations, and of ca. 10.2 °C and ca.11.8 °C for OUT (i.e., outside the tree canopy) in RC and MSG, respectively, ranging from a minimum of −0.6 °C (OUT; 12 January 2012) to a maximum of 29.6 °C (OUT; 12 July 2012) in RC, and a minimum of 0.2 °C (OUT; 23 February 2012) to a maximum of 27.7 °C (OUT; 13 July 2012; Figure 1A) in MSG. The lowest mean temperatures (ca. 1 °C) were detected in the period III in all experimental sites, whereas the highest mean temperatures were reached in the period VI with ca. 18 °C for RC IN and MSG IN, and ca. 22 °C for RC OUT and MSG OUT (Figure 1A). The length of the effective cold stratification periods (i.e., mean daily temperatures < 5 °C) was 92 days for RC IN and MSG IN (both with 41 days of snow cover), and 98 days for RC OUT (with 47 days of snow cover) and 93 days for MSG OUT (with 44 days of snow cover), and occurred from December to March (Figure 1A). The number of days with mean daily temperatures > 20 °C was 64 and 44 days for RC IN and MSG IN, respectively, and 80 days for RC OUT and 96 days for MSG OUT, and occurred from June to August-early September (Figure 1A).

Embryos of *A. barbaricina* seeds (with initial embryo length of ca. 0.3 mm; Table 1) grew slowly from July 2011 (date of field sowing) to December 2011 (period II; Figure 1B). In March 2012 (period IV) embryos started to grow rapidly and reached an embryo length of ca. 1.1 mm both in RC IN and OUT; at the same time a few seeds (ca. 30%) had started to germinate in RC IN (Figure 1B,C). In April 2012, between period IV and V, the seeds reached their critical embryo length (ca. 1.2 mm) and the majority of the seeds had germinated, reaching values of approx. 80% both in RC IN and OUT (Figure 1B,C). In June 2012 (period VI), the percentage of germinated seeds of *A. barbaricina* was ca. 95% both IN and OUT RC experimental sites (Figure 1C). More specifically, both critical embryo length and maximum germination were recorded in April (Figure 1B,C), however, considering that this species is characterised by a multi-step seed germination with significant overlap among all the phases [19], it is likely that the critical embryo length may be also reached before radicle protrusion.

Embryo length in *P. corsica* seed did not change from the initial value (ca. 1.4 mm; Table 1) from the date of sowing (September 2011) to June 2012 (period VI; Figure 1B). By September 2012 (period I), the embryo approximately doubled in length, reaching 2.6 and 3.4 mm for RC IN and OUT, respectively (Figure 1B). By this time, ca. 10% and 56% of seeds for RC IN and OUT, respectively had germinated (Figure 1C). Critical embryo length (ca. 4 mm) was reached at the end of December 2012, between the end of period II and the start of period III (i.e., start of cold stratification period), and 78% and 50% of seeds germinated in RC IN in RC OUT, respectively (Figure 1B,C). At this exhumation time, no seeds in RC IN and 30% of seeds in RC OUT had emerged epicotyls (Figure 1D). At the last exhumation, in April 2013 (period IV), emerged epicotyls were ca. 45 and 70% for RC IN and OUT, respectively (Figure 1D). Each phase of seed germination in *P. corsica* occurred in the second year after sowing. The critical embryo length was reached during September to December (depending on the site position), seeds germinated in December in RC IN and in September in RC OUT, but epicotyls emerged in April in both experimental sites (Figure 1).

Seed germination in *R. sandalioticum* was faster than the other two species. From the date of field sowing (September 2011) to December 2011 (period II) the embryo grew from an initial length of ca. 0.5 mm (Table 1) to around the critical embryo length (ca. 1.8 mm), and the seeds germinated to 58% in RC IN and 84% in RC OUT; but no seeds had emerged epicotyls (Figure 1B–D). To summarize, the critical embryo length in *R. sandalioticum* seeds was reached in December, and at the same time the seeds germinated, while epicotyl emergence occurred in March (Figure 1).

Generalized linear models (GLM) identified a high statistically significant (*p* < 0.001) effect for all three factors (“Date of exhumation”, D; “Position”, P; “Species”, S; Supplementary Table S1) for embryo length. For seed germination and epicotyl emergence, GLMs highlighted a high statistically significant difference (*p* < 0.001) for the “D” and “S” factors and a statistically significant (*p* < 0.05) effect for the “P” factor (Supplementary Table S1). A highly significant difference (*p* < 0.001) was found for all the two-way interactions (D × P, D × S, and P × S) on embryo length, seed germination and epicotyl emergence (Table S1). No significant differences (*p* > 0.05) were detected for the three-way interaction (D × P × S) for embryo length, seed germination, and epicotyl emergence (Supplementary Table S1).

### 2.2. Soil Heat Sum for Embryo Growth and Seed Germination of Aquilegia barbaricina

**Figure 2:**
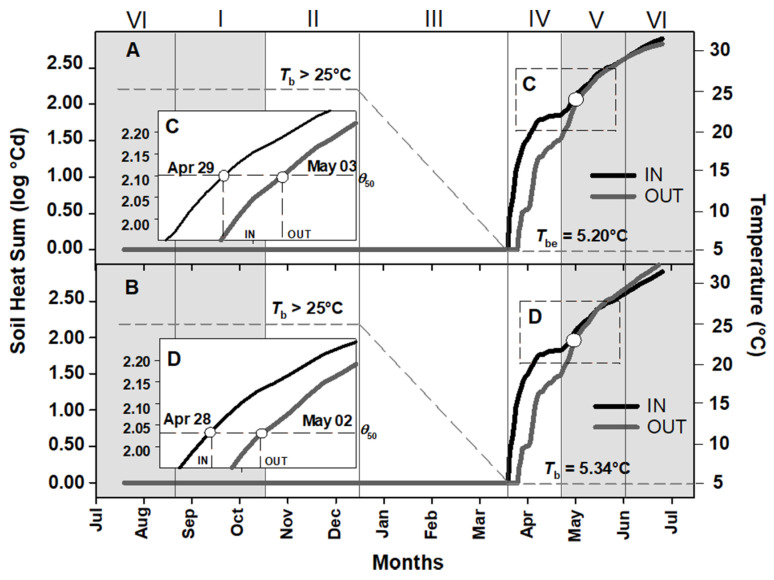
Soil Heat Sum (expressed in log °Cd) to achieve the *θ*_50_ threshold value for (**A**) embryo and (**B**) germination of *Aquilegia barbaricina*, both underneath (IN) and outside (OUT) the tree canopy. Data are from July 2011 to July 2012. The inset plots (**C**,**D**) show the details of the achievement of the *θ*_50_ threshold value (2.10 and 2.04 log °Cd, for embryo and germination, respectively). Dark grey short dashes represent the base temperature before (*T*_b_ > 25 °C) and after (5.2 and 5.3 °C for embryo growth and seed germination, respectively) cold stratification. The background grey squares correspond to the presence of the tree canopy. I, II, III, IV and V correspond to different periods.

To calculate soil heat sum accumulation for embryo growth and germination of *A. barbaricina*, the estimated thermal threshold values obtained previously in controlled conditions were used [19]. Base temperature for embryo growth *(T*_be_) in non-dormant seeds of this species was 5.2 °C, and a base temperature for germination (*T*_b_) of 5.3°C (Table 1). *T*_b_ > 25 °C was assumed for dormant seeds of this species (Table 1). *θ*_50_ threshold values for embryo growth and seed germination (i.e. the value to achieve 50% of seeds that reached the critical embryo length and 50% of germination in controlled conditions) were 2.10 log °Cd and 2.04 log °Cd, respectively [19]. Figure 2 shows the soil heat sum accumulation until the achievement of *θ*_50_ threshold value for embryo growth (Figure 2A) and germination (Figure 2B) in the field for *A. barbaricina* seeds, both IN and OUT of the tree canopy, according to field germination and temperatures recorded by each data logger. Immediately after sowing (period VI), and during periods I, II, and III, *T*_b_ of dormant seed of *A. barbaricina* was higher than the mean soil temperatures, and this prevented the soil heat sum accumulation both for embryo growth and germination (Figure 2). However, after cold stratification (period III), when the seed dormancy was broken, the lower *T*_b_ values and the increasing soil temperatures allowed the threshold of 2.10 log °Cd (for embryo growth) and 2.04 log °Cd (for germination) to be reached from late April to early May (period V; Figure 2A,B). More specifically, *θ*_50_ for embryo growth was reached on 29 April for IN and 03 May for OUT (287 and 291 days after sowing for IN and OUT, respectively; Figure 2C), while *θ*_50_ for germination was reached on 28 April for IN and 02 May for OUT (286 and 289 days after sowing for IN and OUT, respectively; Figure 2D). This estimated time was confirmed by the embryo measurements and germination recorded in the field (see Figure 1).

### 2.3. Soil Heat Sum Estimates for Seed Germination of Paeonia corsica and Ribes sandalioticum

**Figure 3:**
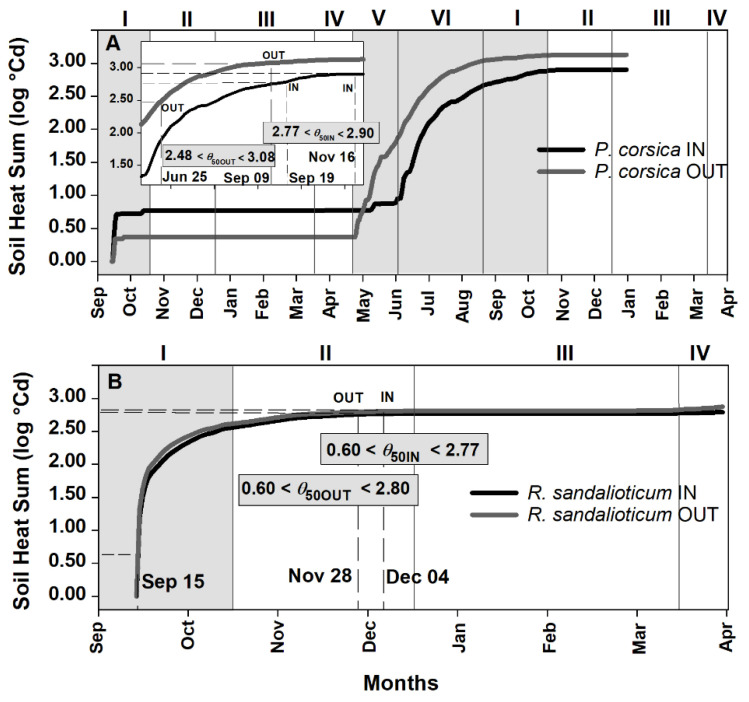
Soil Heat Sum (log °Cd) for the achievement of the predicted minimum and maximum values for germination near to *θ*_50_ threshold value (50% of seed germination in the field) of (**A**) *Paeonia corsica* and (**B**) *Ribes sandalioticum*, both underneath (IN) and outside (OUT) the tree canopy, calculated according to their exhumation times. Data are from September 2011 to January 2013 and form September 2011 to April 2012 for *P. corsica* and *R. sandalioticum*, respectively. The background grey squares correspond to the presence of the tree canopy. I, II, III, IV and V correspond to different periods.

In controlled conditions, non-dormant seeds of *P. corsica* and *R. sandalioticum* germinated only at two of the tested temperatures, namely 10 °C and 15 °C [43,44]. The dataset was not large enough to correlate germination rate and temperature for germination for both species and, consequently, it was not possible to build a complete thermal time model to calculate their *T*_b_, unlike for *A. barbaricina* seeds. For these two species, *T*_b_s were estimated using the lowest tested temperatures at which germination was recorded. These values were 10 °C for non-dormant seed of *P. corsica*, and 5 °C for non-dormant seed of *R. sandalioticum* (see Table 1). Therefore, the soil heat sum accumulation range to achieve the *θ*_50_ threshold value, both IN and OUT (Figure 3), was estimated from the thermal thresholds calculated under laboratory controlled conditions (Table 1) and according to field seed germination percentages obtained during different exhumation times (Figure 1C) combined with the temperatures recorded by each data logger (Figure 1A). From the date of sowing (September; period I) to the end of period V (June), seeds of *P. corsica* were not exposed to warm temperature (i.e. period VI) and *T*_b_ estimated for dormant seeds was higher than the mean soil temperatures; and this prevented the soil heat sum accumulation (Figure 3A). The increasing soil temperatures during period VI allowed the beginning of soil heat sum accumulation. During this period, seeds of *P. corsica* released PD dormancy and the *T*_b_ estimate decreased to a value of 10 °C. Consequently, the rate of soil heat sum accumulation increased. The absence of germination (0%) observed in June and the germination obtained in September in RC OUT (ca. 56%) allowed us to estimate that the *θ*_50_ values for seed germination were within the range of 2.48 log °Cd–3.08 log °Cd in this experimental site. In contrast, the germination of ca. 10% and ca. 76 % recorded in RC IN in September and in December, respectively (see Figure 1C), indicated that the *θ*_50_ estimates were within the range 2.77 log °Cd–2.90 log °Cd (Figure 3A). And the value of 2.90 log °Cd was reached on 16 November. 

As regards *R. sandalioticum* (Figure 3B), the *T*_b_ estimated for dormant and non-dormant seeds of this species (Table 1) was lower than the mean soil temperatures, and this promoted soil heat sum accumulation during September (period I). During the first exhumation carried out in December (period II), seed germination was > 50% in both experimental sites of MSG (see Figure 1C). By this time, seeds had accumulated 2.77 log °Cd in MSG IN and 2.80 log °Cd in MSG OUT (Figure 3B).

### 2.4. Seed Germination Phenology under Different Climate Scenarios

**Figure 4:**
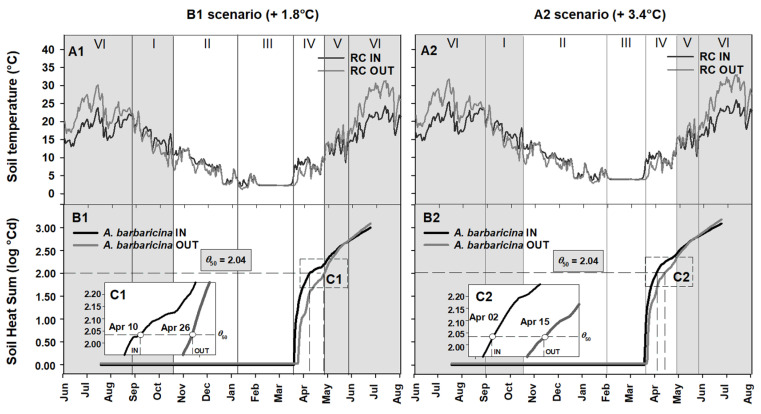
(**A1**,**A2**) Soil temperatures for Rio Correboi (RC) and (**B1**,**B2**) Soil Heat Sum (expressed in log °Cd) to achieve the *θ*_50_ threshold value (2.04 log °Cd) for *Aquilegia barbaricina* seed germination, both underneath (IN) and outside (OUT) the tree canopy, under two different Intergovernmental Panel on Climate Change (IPCC) scenarios (B1, +1.8 °C and A2, +3.4 °C). The inset plots (**C1**,**C2**) show the details of the achievement of the *θ*_50_ threshold value. The background grey squares correspond to the presence of the tree canopy. I, II, III, IV, V and VI correspond to different periods.

**Figure 5:**
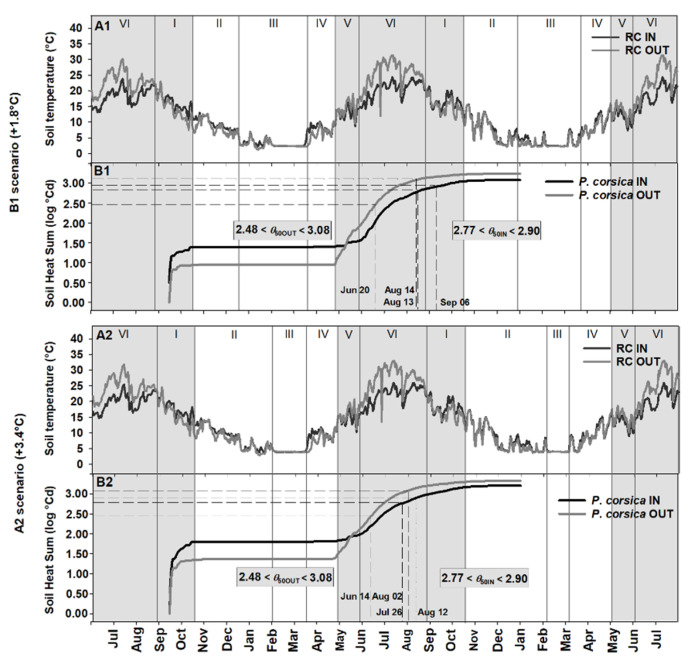
(**A1**,**A2**) Soil temperatures for Rio Correboi (RC), and (**B1**,**B2**) Soil Heat Sum (log °Cd) to achieve the predicted minimum and maximum values for germination near to the *θ*_50_ threshold value for *Paeonia corsica*, both underneath (IN) and outside (OUT) the tree canopy, under two different Intergovernmental Panel on Climate Change (IPCC) scenarios (B1, +1.8 °C and A2, +3.4 °C). The background grey squares correspond to the presence of the tree canopy. I, II, III, IV, V and VI correspond to different periods.

**Figure 6:**
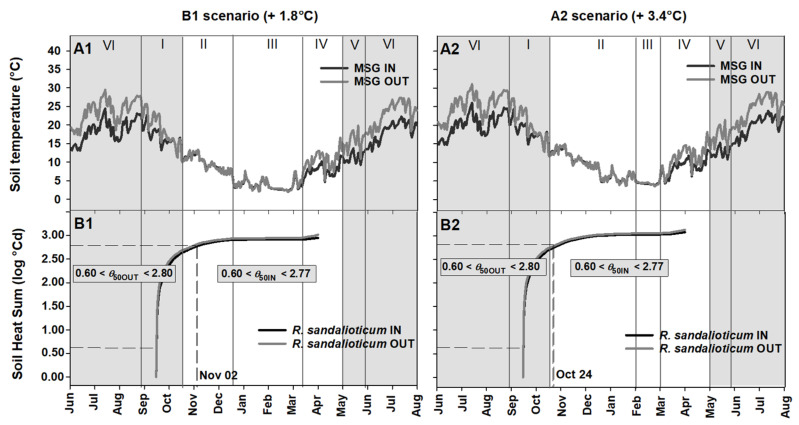
(**A1**,**A2**) Soil temperatures for Monte Novo San Giovanni (MSG), and (**B1**,**B2**) Soil Heat Sum (log °Cd) to achieve the predicted *θ*_50_ threshold value for *Ribes sandalioticum*, both underneath (IN) and outside (OUT) the tree canopy, under two different Intergovernmental Panel on Climate Change (IPCC) scenarios (B1, +1.8 °C and A2, +3.4 °C). The background grey squares correspond to the presence of the tree canopy. I, II, III, IV, V and VI correspond to different periods.

Figure 4, Figure 5 and Figure 6 show the soil heat sum accumulation and the achievement of the *θ*_50_ of each species in the field, both IN and OUT, under two different IPCC scenarios (B1, +1.8 °C and A2, +3.4 °C). Figure 4 shows the soil heat sum accumulation and the achievement of the *θ*_50_ threshold value for *A. barbaricina*. The increase in temperature of +1.8 °C (B1 scenario) and +3.4 °C (A2 scenario) in RC should lead to a reduction of period III (i.e., cold stratification) from ca. 90 days in B1 scenario (Figure 4A1) to ca. 45 days in A2 scenario (Figure 4A2), with an increase of the mean soil temperature of ca. 3 °C in the latter scenario (Figure 4A2) with respect to the present mean soil temperature (ca. 1 °C; Figure 1A). The increase in the mean temperature during period III would not compromise seed dormancy release in *A. barbaricina*. However, after the cold stratification period, the increased temperature would accelerate germination of non-dormant seeds, bringing it forward from late-April to middle-April in RC IN and from early-May to late-April in RC OUT for the B1 scenario (Figure 4B1,C1), and to early-April and middle-April in RC IN and RC OUT, respectively, for the A2 scenario (Figure 4B2,C2).

The mean soil temperature for RC IN should increase from approx. 19 °C to approx. 21 and 23 °C in B1 and A2 scenarios, respectively; while for RC OUT it should increase from approx. 24 °C to 26 and 28 °C in B1 and A2 scenario, respectively (Figure 5A1,A2). In particular, an increased soil heat sum would accelerate the achievement of the *θ*_50_ threshold value for seed germination in *P. corsica*, both in RC IN and RC OUT (Figure 5B1,B2). The increase in temperature predicted in B1 scenario could bring forward the favourable period for seed germination of this species to June-August for RC OUT and to August-September for RC IN (Figure 5B1). Similarly, the increase in temperature predicted in the A2 scenario could bring forward the best time for seed germination to June-early August for RC OUT and to July-August for RC IN (Figure 5B2). Moreover, in these latter scenarios, the seed germination of this species would coincide mainly with the summer drought period (period VI; Figure 5A2,B2), i.e., in a period with no or sporadic rainfall, a condition that may compromise the germination of seeds of *P. corsica* in RC OUT, where soil moisture would be less than under shade (i.e., absence of tree canopy) as in RC IN.

An increased soil heat sum would accelerate the achievement of the *θ*_50_ threshold value in *R. sandalioticum* seeds (Figure 6B1,B2). In the B1 scenario, seed germination should advance to November (Figure 6A1,B1), while in the A2 scenario it could happen in October (Figure 6A2,B2). More specifically, seed germination of this species would occur ca. 38 and 45 days earlier in the B1 and A2 scenarios, respectively (Figure 6B1,B2).

## 3. Discussion

### 3.1. Ecological Correlates of Embryo Growth, Seed Germination, Epicotyl Emergence and Seedling Establishment in Natural Conditions

The phenology of embryo growth, radicle, and epicotyl emergence were analysed in seeds of three mountain species (*A. barbaricina*, *P. corsica,* and *R. sandalioticum*) occupying similar habitats. All three species have endospermic seeds that are reputed to have MPD. In addition, seeds of *P. corsica* and *R. sandalioticum* have been shown to have epicotyl dormancy [43,44]. The seed embryos are small at dispersal and must grow before radicle emergence. As detected in controlled conditions, the seeds of these species require specific temperature treatment to overcome their morphophysiological dormancy [19,43,44]. However, in their natural habitats, these taxa responded differently to temperature and this impacts the timing of in situ germination. All studied species disperse seeds between period VI and I, in the presence of the tree canopy. Thereafter, seeds have increased access to water through higher rainfall. Nonetheless, the three species have developed subtle differences in interpreting temperature so that their seed germination strategies to avoid unfavourable environmental conditions for plant establishment vary. In detail, seeds of *A. barbaricina* are dispersed in summer [19] and germinate the following spring/early summer in the presence of tree canopy. During the summer and early autumn, the embryos grow slowly, and low temperature during autumn/winter (mean soil temperature ≤ 5 °C) facilitates cold stratification and the release of physiological dormancy. Germination ensues after winter when mean soil temperatures reach at least 10–15 °C. From our understanding of the seed and embryo response to temperature, once dormancy is broken through cold treatment the embryos start to grow more rapidly inside the seeds; and once the critical embryo length is reached, germination completion (i.e., radicle emergence) happens quickly. Thus, the multi-step process of germination in this species [19] ensures that the seeds germinate during the early spring and the seedlings can grow before the dry summer period.

Seeds of *P. corsica* are dispersed in late summer/early autumn in the presence of the tree canopy, and are exposed to a mean soil temperature near and below 20 °C. However, the seeds stay dormant until the following summer when they are then exposed to warm temperatures (mean soil temperature > 20 °C), during which the length of the embryo remains stable. During the following late summer/early autumn, when the temperature falls to 10 °C–15 °C and the rainfall increases, the embryos grow inside the seeds; a doubling in embryo length is necessary before radicle emergence is possible. The critical embryo length and radicle protrusion are reached in late autumn before the natural cold stratification period but after that the seeds accumulate warm temperature during summer, consistent with the multi-step seed germination observed in controlled conditions [44]. Seed germination starts in the presence of tree canopy and is completed in December when the canopy is absent. Germinated seeds go through the winter with an emerged radicle, and the cold stratification period allows epicotyls to emerge in the following April when mean soil temperatures reach at least 10 °C–15 °C. Seedlings with well-developed roots and shoots are established before the end of the second wet season and before the dry summer period when the canopy is absent. Successively, the young plants growing underneath of tree canopy may benefit from the closure of the canopy during the summer.

Berries of *R. sandalioticum* are dispersed in late summer (mainly by birds and ⁄ or mammals, although many fruits simply drop to the ground). After dispersal, the seeds of *R. sandalioticum* are exposed immediately to mean soil temperatures near to 20 °C [43]. During this time, seed temperature facilitates removal of MPD by promoting embryo growth. Subsequent seed germination, from September onwards, is enabled by increased availability of water through higher rainfall. Embryos start to grow inside the seeds from November to December, likely when mean soil temperatures drop below 15 °C. Germinated seeds of *R. sandalioticum* go through the winter with an emerged radicle and are exposed to cold temperature (mean soil temperature ≤ 5 °C) during December to March. In spring, when mean soil temperatures again reaches 10–15 °C, the epicotyls emerge, and the seedlings establish before the end of the wet season and prior to the start of the summer. 

Seed germination of *A. barbaricina* and *R. sandalioticum* occurs during periods IV and II, respectively; therefore, the seed germination for these two species seems not be linked to the presence of the tree canopy. A similar pattern was found in *Rhamnus persicifolia*, a species that grows in the same ecosystem and ecological conditions in Sardinia [26]. In *P. corsica*, on the contrary, the tree canopy seems to have a negative influence on seed germination. Maximum germination for OUT (i.e. outside the tree canopy) is in September and only a few germinated seeds appear for IN in this period, while the maximum germination for IN is in December when the canopy is absent. In all species, however, closure of the tree canopy could influence survival of newly established seedlings due to microclimate amelioration (moister and cooler) during the dry and hot Mediterranean summers [41,45,46]. The seeds of the all the investigated species showed a high synchronisation with the Mediterranean seasonality with respect to embryo growth, seed germination and seedling establishment, and thus demonstrate particular adaptations to the harsh Mediterranean climatic conditions.

### 3.2. Soil Heat Sum for In Situ Seed Germination

The quantification of thermal time for germination has been used in different studies to characterize changes in seed dormancy and subsequent germination in the field (i.e., [20,22,26,47]). Although complex, such modelling can be used to connect laboratory and field studies. Here, we used the soil heat sum model [26], and the thermal threshold (*θ*_50_) of *A. barbaricina* estimated in the laboratory [19] to predict embryo growth and seed germination phenology in the field. We observed a high correlation between soil heat sum accumulation to reach *θ*_50_ for the critical embryo length and seed germination. The model showed that, in the original population, these values are reached between April and May, thus confirming that the *θ*_50_ for embryo growth and the *θ*_50_ for seed germination of *A. barbaricina* approximately coincide. Results were validated through field observations of embryo growth measurements and seed germination. The model can also be used to estimate the range of *θ*_50_ for seed germination in species where the thermal time value (*θ*_50_) in controlled conditions is unknown. By evaluating the seed germination behaviour in field conditions and by using the recorded soil temperatures, the model generated estimates for soil heat sum accumulation for seed germination of *P. corsica* and *R. sandalioticum* seeds and an approximation of seed thermal requirements. The estimated *θ*_50_ values for seed germination of *P. corsica* fall within the range of 2.48–2.90 log °Cd, while for *R. sandalioticum θ*_50_ values for seed germination are estimated around 2.70 log °Cd. One benefit of knowing the thermal requirements for each species is being able to predict the seed germination phenology under increasing temperatures due to global warming.

### 3.3. Phenology of Seed Germination under Global Warming

Results of this study highlighted that a uniform increase of temperature under B1 (+1.8 °C) and A2 (+3.4 °C) scenarios would affect the rate of seed heat sum accumulation. In particular, soil heat sum under these two different IPCC scenarios bring completion of germination forward by about one month for all three studied species. As reported by Mondoni et al. [48], climate warming could lead to a shift of the timing of seed germination but the extent of this change across species will be driven by seed dormancy status. Porceddu et al. [26] indicated that the warmer temperatures predicted by two simulated IPCC scenarios may reduce the cold stratification period useful for dormancy release in *R. persicifolia* seeds; however, the increasing temperatures and the consequent reduction of the stratification period would not be detrimental per se for seed germination. Similarly, an increase in soil temperature may alter the timing of germination of *Polaskia chende* and *P. chichipe* but this will not be detrimental for germination success [27]. In addition, a decrease in the length of winter due to global warming may not affect seed dormancy release, e.g., in *Halenia elliptica* D. Don [49]. Contrary to these responses, the future conditions may not meet the requirements for breaking physiological dormancy in *Gentiana lutea* subsp. *lutea* [24] and *Vitis vinifera* subsp. *sylvestris* [23] seeds and will be detrimental to the proportion of seeds which germinate. The increasing temperature predicted in both scenarios tested in this study might not compromise the dormancy release of *A. barbaricina*; however, as seeds of this species need a cold stratification period to promote dormancy release, further research is recommended to understand the seed germination behaviour under a probable cold stratification reduction, as previously done for *R. persicifolia* [26] and *G. lutea* subsp. *lutea* [24]. In addition, the phenological shift of seed germination could enhance the seedling growth of this species before the harsh conditions set in. On the contrary, this phenological shift, in particular in A2 scenario, could increase the risk of late frosts in spring which could damage young seedlings and increase the potential mortality of plants. Dormant seeds requiring warm stratification may exhibit little difference in dormancy release if moisture increases, but they may not lose dormancy if soil moisture decreases [33]. The rate of dormancy loss may vary due to the temperature rise and germination could be triggered outside normal wet seasons leading to lowered seedling survivorship during the dry conditions [33]. The increasing temperature might be a disadvantage for *P. corsica*, as some seed germination could occur during the summer when there are short episodes of rainfall. However, these are interspersed by drought, and the risk of mortality for young seedlings would also increase. Closure of the canopy in the riparian woods could counteract some of this risk. Microclimate amelioration (moister and cooler) under shade, even during the dry and hot Mediterranean summers, would mean less evaporation of water from soil and the newly established seedlings might survive better. The bringing forward of seed germination in *R. sandalioticum* would not cause particular problems for the seedlings growth because it would coincide with the period of maximum rainfall and mild temperatures. However, the sensitivity of *R. sandalioticum* to low temperatures for seed germination highlights the presence of an increasing threat from global warming. In fact, as detected in laboratory conditions, embryo growth and seed germination occur only at 10°C and 15°C [43]. This narrow temperature requirement for seed germination of *R. sandalioticum* could reduce the level of natural emergence in the field [43]. Similar behaviours have also been identified for the only congeneric species, *Ribes sardoum* Martelli, which is also present in Sardinia [50]. However, while the results of this study are based on the assumption that the thermal thresholds for seed germination will remain constant trough climate change, further studies should be carried out on these species to understand how climate change might affect the successive steps of seed production, dispersal, dormancy, and germination, as they also have a thermal memory (via phenotypic plasticity) that incorporates information from past thermal history [51].

In summary, we have provided a detailed explanation of the ecophysiology of seed germination in three Mediterranean mountain species with morphophysiological dormancy. Moreover, through the quantification of seed germination, we have been able to provide an important means of connecting the micro-environmental niche for in situ seed germination of the species, both under and outside a tree canopy, and the macro-environmental parameters under various global warming scenarios. 

## 4. Materials and Methods

### 4.1. Study Species

**Figure 7:**
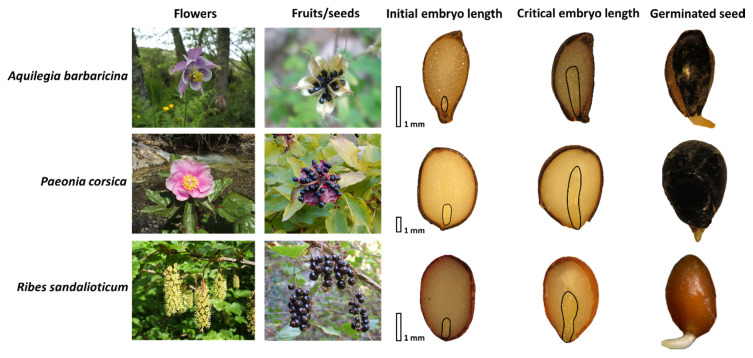
Flowers, fruits/seeds, initial and critical embryo length, and germinated seed of three endospermic species studied: *Aquilegia barbaricina*, *Paeonia corsica* and *Ribes sandalioticum*.

*Aquilegia barbaricina* (Ranunculaceae), *Paeonia corsica* (Paeoniaceae) and *Ribes* *sandalioticum* (Grossulariaceae) (Figure 7) are endemic species of Sardinia and grow frequently in the same localities from ca. 1000 m a.s.l. to the higher elevation of CE-Sardinia mountains, in wet woodlands, meadows and stream margins under and near riparian woods (see Table 1 and Supplementary Table S2). In detail, *A. barbaricina* is endemic to the Gennargentu and Supramontes regions where the plant grows from 800 to 1400 m a.s.l. [52]. *P. corsica* is endemic of Sardinia and Corsica, and in Sardinia, it grows from 600 to 1700 m a.s.l. [53]. *R. sandalioticum* is found in small populations in the Supramontes, Gennargentu Massif, Catena del Marghine and Limbara Mountain, growing at altitudes above 1000 m a.s.l. [54]. The seeds of these species are endospermic and the embryos are linear, underdeveloped at dispersal, and need to grow to a critical length before radicle emergence (see Figure 7).

Information of initial (i.e., the length of the embryo at dispersal) and critical (i.e., the length of the embryo in seeds with a split seed coat but no radicle protrusion) embryo length and seed germination obtained in controlled conditions were taken from previous works and are reported in Table 1.

### 4.2. Seed Lot Details

**Figure 8:**
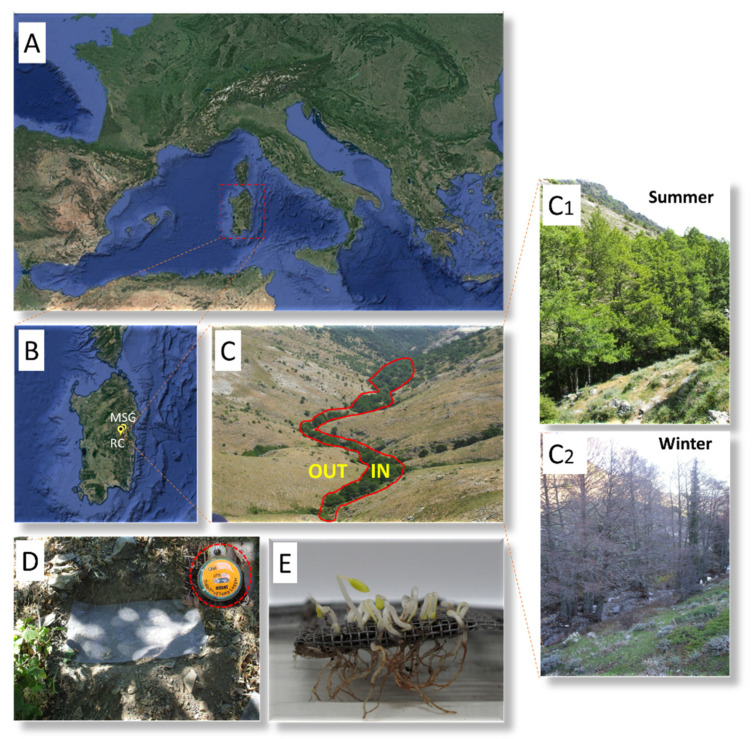
(**A**) Geographical location of Sardinia in the Mediterranean context, (**B**) localisation of Monte Novo San Giovanni (MSG) and Rio Correboi (RC), (**C**) representation of the experimental sites underneath (IN) and outside (OUT) the tree canopy characterised by (**C1**) the presence or (**C2**) absence of canopy during the year, (**D**) envelops and data logger buried in the soil and (**E**) example of retrieved envelope with germinated seeds.

Seeds of *A. barbaricina*, *P. corsica* and ripe berries of *R. sandalioticum* were collected directly from plants at the time of natural dispersal in 2011 in their representative natural populations, named Rio Correboi (RC) and Monte Novo San Giovanni (MSG) (Table 1; Figure 8; Supplementary Table S2). Seeds of *R. sandalioticum* were immediately separated from the pulp by rubbing fruits through sieves under running water. The cleaned seeds were then spread out and left to dry at room temperature. Seeds of all species were manually cleaned, and well-developed seeds were selected in the laboratory, discarding any visually malformed seeds, and maintained at room temperature (ca. 40% of relative humidity and 20 °C) until the start of the in situ experiments.

### 4.3. Seed Germination and Embryo Growth in Natural Conditions

According to the methodology in Porceddu et al. [26], seeds of each species were placed in fine-mesh polyester envelopes (3 replicates of 25 seeds) and buried in the soil at a depth of 2–3 cm, within ca. 20 days after seed collection (Figure 8; Supplementary Table S2). Envelopes were buried both underneath (IN) and outside (OUT) the tree canopy (Figure 8), with a distance between them of ca. 6 m, in each natural population (Supplementary Table S2). Envelopes buried in the experimental sites were exhumed at approximately three-month intervals from September 2011 to June 2012 (with an intermediate exhumation also in April 2012) for *A. barbaricina*, from September 2011 to March 2012 for *R. sandalioticum*, and from September 2011 to December 2012 for *P. corsica.* A further exhumation for *P. corsica* was also performed in March 2013 to evaluate the number of seeds with epicotyl-plumule (hereafter epicotyl) emerged.

Retrieved envelopes were analysed in the laboratories of the Sardinian Germplasm Bank (BG-SAR) [55], where they were washed under running water and opened. The number of germinated and epicotyl emerged seeds was recorded. In addition, embryo growth in the field was assessed during each exhumation time (Supplementary Table S2), by measuring 20 randomly chosen seeds among all seeds within the three replicates. Seeds were cut in half under a dissecting microscope and images of embryos were acquired using a Zeiss SteREO Discovery.V8, with an objective Achromat S 0.63x, FWD 107mm (Carl Zeiss MicroImaging GmbH) at a 6.3× magnification for *A. barbaricina* and *R. sandalioticum* and at a 4.0× magnification for *P. corsica*, coupled to a Canon (Power shot G11) digital camera. Embryo and seed lengths were measured using the image analysis software ImageJ 1.41ᴏ (National Institutes of Health, Bethesda, MA, USA). Seed length was measured ignoring the seed coat. The critical embryo lengths reported in Table 1 were used for seeds that had germinated before measurements [56].

### 4.4. Soil Heat Sum Approach

A soil heat sum approach was used to predict field germination phenology for all the investigated species according to [26]. Soil temperatures at the level of the envelopes were recorded both underneath (IN) and outside (OUT) the tree canopy of the natural population sites at 90-minutes intervals, using data loggers (TidbiT^®^ v2 Temp logger, Onset Computer Corporation, Cape Cod, Massachusetts, U.S.). Soil temperatures above *T*_b_ of each species (Table 1) were used to assess the temperature accumulation till the achievement of the thermal time required for 50% germination (*θ*_50_). Soil heat sum was calculated, starting from the date of sowing (Supplementary Table S2), according to the following equation (Equation (1)):

*Soil heat sum* (log °Cd) = {∑ [(*T*_S_ − *T*_b_) x *t*]}/16, (1)

where *T*_S_ is the temperature at each logging interval recorded by data loggers, *T*_b_ is the base temperature for seed germination of each species (see Table 1), *t* is the length of the logging interval expressed in hours and 16 is the number of logging records per day [26].

In addition, soil temperatures recorded by data loggers at each logging interval (*T*_S_) above *T*_be_ (i.e., the base temperatures for embryo growth) of *A. barbaricina* [19] were used to estimate the soil heat sum accumulation till the achievement of the thermal times to reach 50% of seeds that reached the critical embryo length [19] according to the following equation (Equation (2)):

*Soil heat sum* (log °Cd) = {∑ [(*T*_S_ − *T*_be_) x *t*]}/16. (2)

Pluviometric data for Rio Correboi (monthly averages of rainfall from 1922 to 2009 from the nearby climatic station of Fonni, NU) and Monte Novo San Giovanni (monthly averages of rainfall from 1936 to 2009 from the nearby climatic station of Montes, Orgosolo, NU), were acquired from Regione Autonoma della Sardegna (http://www.regione.sardegna.it/j/v/25?s=131338&v=2&c=5650&t=1). The presence/absence of the tree canopy of riparian wood (Figure 8) was observed at each field excursion during this study; according to the seasons and to the presence/absence of the canopy, the different periods were identified [26]. In detail, the different periods correspond to: (I) from late August at the end of September/early October to the disappearance of the tree canopy in mid-October; (II) from the disappearance of the canopy in mid-October to the start of the cold stratification period, when mean daily temperatures fell to 5 °C in December; (III) the main cold stratification period, from December to March, when mean daily temperatures are close to 5 °C; (IV) from the end of the cold stratification period in March to the appearance of the canopy in April; (V) from the appearance of the canopy in April to the start of the summer droughts in June/July; and (VI) the summer drought period when rainfall drastically reduces and the temperatures are high.

Seed germination phenologies under different climate scenarios [28,29] were estimated increasing the soil temperature recorded for each study area according to IPPC scenarios B1 (low emissions scenario, +1.8 °C, which falls within the range of the two intermediate scenarios RCP4.5 and RCP6.0) and A2 (high emission scenario; +3.4 °C; value within the range of the RCP8.5 scenario). Accordingly, the soil heat sum to achievement of the estimated threshold values for seed germination (*θ*_50_) were calculated again according to the previous equation.

### 4.5. Statistical Analysis

Generalized linear models (GLMs) were used to compare the field embryo length, seed germination and epicotyl emergence percentages of each species at different exhumation dates, both IN and OUT the tree canopy. GLM with a log link function and quasipoisson error structure was used for analysing embryo length values, while GLMs with a logit link function and quasibinomial error structure were used when analysing seed germination and epicotyl percentages. Quasibinomial and quasipoisson error structures and *F* tests with an empirical scale parameter instead of chi-squared on the subsequent ANOVA were used to overcome residual overdispersion [57]. All statistical analyses were carried out with R v. 2.14.0 [58].

## 5. Conclusions

In conclusion, the seed germination phenology of three endemic Mediterranean mountain species with endospermic seeds growing in the same ecosystem was shown to vary considerably. Using parameters generated on the thermal requirements for embryo growth and radicle emergence under controlled conditions for *A. barbaricina*, it was possible to predict in situ emergence and the responses were validated through field observations. The results show that it is possible to combine the developed species-specific models with a soil heat sum approach and predict with good accuracy seed germination in the field. Furthermore, this approach and the model developed may have applicability for the prediction of in situ regenerations under different IPCC scenarios of increasing temperatures.

## Figures and Tables

**Table 1:** Published thermal traits and germination characteristics of *A. barbaricina*, *P. corsica* and, *R. sandalioticum*. *T*_b_ and *T*_be_ correspond to the base temperatures for seed germination and for embryo growth, respectively. *θ*e_50_ and *θ*g_50_ correspond to the threshold values for embryo growth and seed germination, respectively. * *T*b values estimated as the lowest tested temperature at which germination occurred (*sensu* [16]).

Species	*A. barbaricina*	*P. corsica*	*R. sandalioticum*
**Population**	Rio Correboi (Villagrande Strisaili, NU)	Rio Correboi (Villagrande Strisaili, NU)	Monte Novo San Giovanni (Orgosolo, NU)
**Maximum Germination in Laboratory (%)**	81 ± 12	63± 10	88 ± 3
***T*_b_ (°C) Dormant Seeds**	> 25	15 *	10 *
***T*_b_ (°C) Non-dormant Seeds**	5.34 ± 1.38	10 *	5 *
**Initial Embryo Length (mm)**	0.29 ± 0.06	1.40 ± 0.20	0.52 ± 0.08
**Critical Embryo Length (mm)**	1.17 ± 0.23	3.90 ± 0.70	1.80 ± 0.39
***T*_be_ (°C)**	5.20 ± 0.60	ND	ND
***θ*e_50_ (log °Cd)**	2.10	ND	ND
***θ*g_50_ (log °Cd)**	2.04	ND	ND
**Source**	[19]	[44]	[43]

## Supplementary Materials

The following are available online at https://www.mdpi.com/2223-7747/9/10/1382/s1, Table S1: GLMs results for the effect on (I) embryo length, (II) seed germination and (III) epicotyl emergence in the field of the following factors: “Date of exhumation” (D: see Table S2), “Position” (P: IN and OUT) and “Species” (S: *A. barbaricina*, *R. sandalioticum* and *P. corsica*). Table S2: Locations, habitat characteristics and dates of experimental trials carried out in each site (Rio Correboi: RC IN and RC OUT; Monte Novo San Giovanni: MSG IN and MSG OUT) of the natural populations of each species. For each experimental site, IN and OUT differentiate between underneath and outside the tree canopy, respectively.
