# Supplementary material for: Differential Interpretation of Mountain Temperatures by Endospermic Seeds of Three Endemic Species Impacts the Timing of In Situ Germination
Source: Plants (Basel). 2020 Oct 16;9(10):1382. doi: 10.3390/plants9101382 (PMC7603068; doi:10.3390/plants9101382)
Supplement: Supplementary file 1 [file plants-09-01382-s001.pdf]

Supplementary materials

Differential interpretation of mountain temperatures by endospermic seeds of three endemic species impacts the timing of *in situ* germination

[1] Marco Porceddu [1,2,*], Hugh W. Pritchard [3], Efisio Mattana [3] and Gianluigi Bacchetta [1,2]

[1] Sardinian Germplasm Bank (BG-SAR), Hortus Botanicus Karalitanus (HBK), University of Cagliari, Viale S. Ignazio da Laconi, 9-11, Cagliari 09123, Italy; porceddu.marco@unica.it, https://orcid.org/0000-0002-3180-9000 (M.P.); bacchet@unica.it, https://orcid.org/0000-0002-1714-3978 (G.B.)

[2] Centre for the Conservation of Biodiversity (CCB), Life and Environmental Sciences Department, University of Cagliari, Viale S. Ignazio da Laconi 11-13, 09123 Cagliari, Italy; (M.P. and G.B)

[3] Royal Botanic Gardens, Kew, Wellcome Trust Millennium Building, Wakehurst, Ardingly, West Sussex RH17 6TN, UK; h.pritchard@kew.org, https://orcid.org/0000-0002-2487-6475 (H.W.P.); E.Mattana@kew.org, https://orcid.org/0000-0001-6235-4603 (E.M)

* Correspondence: porceddu.marco@unica.it (M.P.)

Table S1 - GLMs results for the effect on (I) embryo length, (II) seed germination and (III) epicotyl emergence in the field of the following factors: "Date of exhumation" (D: see Table S1), "Position" (P: IN and OUT) and "Species" (S: *A. barbaricina, R. sandalioticum* and *P. corsica*).

**(I) Embryo**	**Df**	**Deviance**	**Resid. df**	**Resid. dev**	***F***	***P* (>F)**
NULL			557	354.55		
Date (D)	6	222.432	551	132.12	662.8910	< 0.001
Position (P)	1	0.637	550	131.48	11.3866	< 0.001
Species (S)	2	66.182	548	65.30	591.7089	< 0.001
D × P	6	1.569	542	63.73	4.6769	< 0.001
D × S	5	29.825	537	33.91	106.6599	< 0.001
P × S	2	1.033	535	32.87	9.2371	< 0.001
D × P × S	5	0.527	530	32.35	1.8855	> 0.05
**(II) Germination**	**Df**	**Deviance**	**Resid. df**	**Resid. dev**	***F***	***P* (>F)**
NULL			71	6210.7		
D	6	1802.1	65	4408.7	47.4112	< 0.001
P	1	37.4	64	4371.2	5.9072	< 0.05
S	2	3454.5	62	916.8	272.6571	< 0.001
D × P	6	129.5	56	787.3	3.4066	< 0.001
D × S	3	193.8	53	593.5	10.1971	< 0.001
P × S	2	240.8	51	352.7	19.0097	< 0.001
D × P × S	3	14.0	48	338.7	0.7360	> 0.05
**(III) Epicotyl emergence**	**Df**	**Deviance**	**Resid. df**	**Resid. dev**	***F***	***P* (>F)**
NULL			71	5644.2		
D	6	2883.90	65	2760.3	105.5932	< 0.001
P	1	23.25	64	2737.1	5.1079	< 0.05
S	2	2018.71	62	718.3	221.7438	< 0.001
D × P	6	134.13	56	584.2	4.9113	< 0.001
D × S	3	136.81	53	447.4	10.0186	< 0.001
P × S	2	214.37	51	233.0	23.5471	< 0.001
D × P × S	3	0.00	48	233.0	0.0000	> 0.05

Table S2 - Locations, habitat characteristics and dates of experimental trials carried out in each site (Rio Correboi: RC IN and RC OUT; Monte Novo San Giovanni: MSG IN and MSG OUT) of the natural populations of each species. For each experimental site, IN and OUT differentiate between underneath and outside the tree canopy, respectively.

Species	Population	Experimental sites	Habitat	Altitude (m a.s.l.)	Aspect	Date of field sowing	Dates of exhumation and days after sowing
A. barbaricina	Rio Correboi (Villagrande Strisaili, NU)	RC IN	Riparian wood of black alder (*Glechomo-Alnetum glutinosae*) – Mantle shrubs of elm-leaf blackberry (*Pruno-Rubion ulmifolii*)	1267	0	18/07/2011	16/09/2011 (60 days)<br>09/12/2011 (144 days)<br>29/03/2012 (255 days)<br>26/04/2012 (283 days)<br>25/06/2012 (343 days)
		RC OUT	Open grassland of *Carici-Genistetea lobelioidis*		NE		
P. corsica	Rio Correboi (Villagrande Strisaili, NU)	RC IN	Riparian wood of black alder (*Glechomo-Alnetum glutinosae*) – Mantle shrubs of elm-leaf blackberry (*Pruno-Rubion ulmifolii*)	1267	0	16/09/2011	09/12/2011 (84 days)<br>29/03/2012 (195 days)<br>25/06/2012 (283 days)<br>19/09/2012 (369 days)<br>28/12/2012 (469 days)<br>10/04/2013 (572 days)
		RC OUT	Open grassland of *Carici-Genistetea lobelioidis*		NE		
R. sandalioticum	Monte Novo San Giovanni (Orgosolo, NU)	MSG IN	Mantle shrubs of elm-leaf blackberry (*Pruno-Rubion ulmifolii*)	1225	0	16/09/2011	09/12/2011 (84 days)<br>29/03/2012 (195 days)
		MSG OUT	Open grassland of *Carici-Genistetea lobelioidis*		0		
